# Development of an ic-CLEIA for precise detection of 3-CQA in herbs and patent medicines: ensuring quality control and therapeutic efficacy

**DOI:** 10.3389/fnut.2024.1439287

**Published:** 2024-08-21

**Authors:** Longjiang Wu, Mei Dang, Rao Wu, Murtala Bindawa Isah, Xiaoying Zhang

**Affiliations:** ^1^Chinese-German Joint Institute for Natural Product Research, Shaanxi International Cooperation Demonstration Base, Shaanxi University of Technology, Hanzhong, China; ^2^Department of Biological Sciences, Faculty of Science, National University of Singapore, Singapore, Singapore; ^3^Department of Biochemistry, Faculty of Natural and Applied Sciences, Umaru Musa Yar’adua University Katsina, Katsina, Nigeria; ^4^Biomedical Research and Training Centre, Yobe State University, Damaturu, Nigeria; ^5^Centre of Molecular and Environmental Biology, Department of Biology, University of Minho, Braga, Portugal; ^6^Department of Biomedical Sciences, Ontario Veterinary College, University of Guelph, Guelph, ON, Canada

**Keywords:** 3-caffeoylquinic acid (3-CQA), indirect competitive chemiluminescence enzyme immunoassay (ic-CLEIA), monoclonal antibody (mAb), enzyme-linked immunosorbent assay (ELISA), quality control

## Abstract

**Background:**

3-caffeoylquinic acid (3-CQA), a member of the chlorogenic acid family, possesses diverse pharmacological properties, such as scavenging, antioxidant, and antiapoptotic activity, rendering substantial value to alimentary consumables and therapeutic substances. However, the pervasiveness of non-standard practices, notably the misuse and abuse of indigenous botanicals, coupled with the inherent susceptibility of 3-CQA to degradation under light and heat exposure, engenders discernible disparateness in the quality profiles of the same kinds of herbs. Consequently, precise quantification of 3-CQA becomes imperative.

**Methods:**

In this context, an artificial antigen was synthesized as a specific conjugate of 3-CQA and bovine serum albumin (3-CQA-BSA), followed by the generation of a monoclonal antibody (mAb) against the conjugate. Through optimization, a mAb-based indirect competitive chemiluminescence enzyme immunoassay (ic-CLEIA) was developed.

**Results:**

It demonstrated an IC_50_ and the calibration range of 2.97 ng/mL and 0.64–13.75 ng/mL, respectively, outperforming the conventional enzyme-linked immunosorbent assay (ELISA). Notably, the ic-CLEIA displayed 10.71% cross-reactivity with 3,5-dicaffeoylquinic acid, alongside minimal cross-reactivity toward other isomeric counterparts and analogs. Validation experiments on herbs and Chinese patent medicines using ic-CLEIA, confirmed by high-performance liquid chromatography (HPLC) analysis, revealed a robust correlation coefficient of 0.9667 between the two modalities.

**Conclusion:**

These findings unequivocally demonstrated that the proposed ic-CLEIA represents a viable and reliable analytical method for 3-CQA determination. This method holds significant potential for ensuring the quality control and therapeutic efficacy germane to herbs and patent medicines, spanning diverse therapeutic milieus and applications.

## 1 Introduction

Phenolic compounds, as secondary metabolites of plants, have garnered significant attention in recent years due to their remarkable bioactivity (1–3). Among these compounds, chlorogenic acids (CGAs), a class of phenolic acids, have particularly attracted interest owing to their antioxidative and free radical-scavenging properties (4, 5). CGAs comprise a family of esters derived from quinic acid and specific trans-cinnamic acids, predominantly caffeic, p-coumaric, and ferulic acid. Within the CGA family, although the quantification of 5-caffeoylquinic acid (5-CQA) has been extensively reported (6, 7), limited research exists on its structural isomers like 3-caffeoylquinic acid (3-CQA) and 4-caffeoylquinic acid (4-CQA).

**Figure 1:**
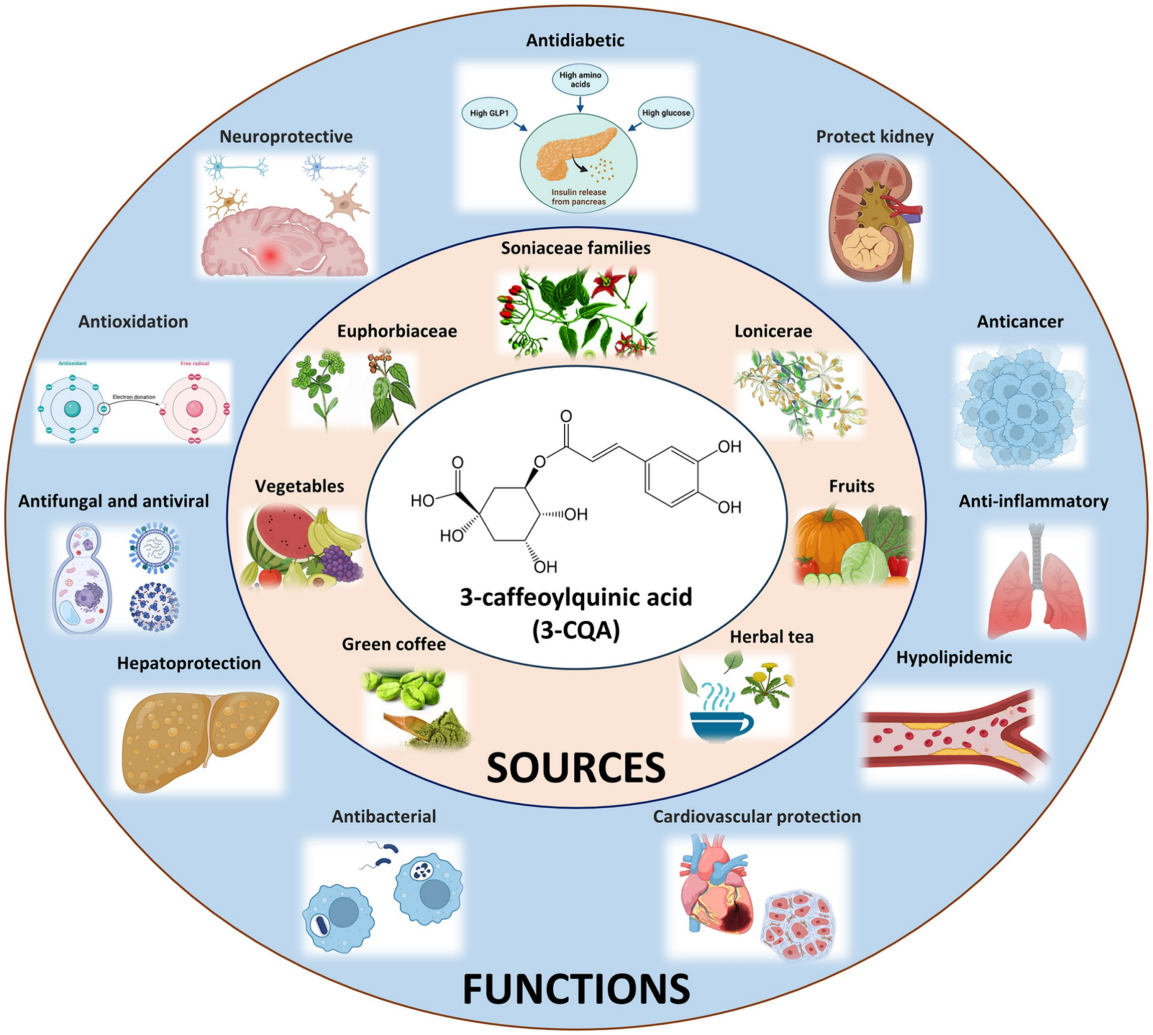
Origins and Bioactive Impacts of 3-CQA. The chemical structure of 3-CQA is represented within the white circle, sources of 3-CQA are delineated within the pink circle, and the bioactive effects of 3-CQA are elucidated within the blue circle.

Considering this, this study focuses on 3-CQA, an ester of caffeic acid and (−)-quinic acid. On the one hand, 3-CQA displays intriguing biological effects, low toxicity, and promising commercial value. Notably, it acts as the primary active ingredient in *Honeysuckle* and *Euphorbia*, demonstrating antibacterial, antiviral, antioxidant, anti-inflammatory, hypolipidemic, and neuroprotective effects (Figure 1) (8–11). Recently, it has been reported to alleviate and prevent diabetes mellitus and associated complications such as diabetic nephropathy and diabetic retinopathy (12). In addition, 3-CQA has shown promise in countering high-fat diet (HFD)-induced activation of the TLR4 signaling pathway and the expression of *TNF-α* and *IL-6* in the liver, thus ameliorating non-alcoholic fatty liver disease (NAFLD) (13). On the other hand, beyond its presence in *Honeysuckle* and *Euphorbia*, 3-CQA is widely distributed in other plants of the *Euphorbiaceae*, *Lonicerae*, and *Soniaceae* families, as well as in various vegetables and fruits (Figure 1) (14–17). However, the prevalence of non-standard practices, along with the vulnerability of the compound *per se* to degradation from light and heat, often leads to considerable variations and challenges in quality control.

Given the impressive pharmacological activities and the scarcity of quantification studies, there is an imperative demand for the development of analytical methods for 3-CQA-containing herbs and associated products. In this context, we capitalized on the intrinsic advantages of canonical chemiluminescence enzyme-linked immunosorbent assay (CLEIA) over other instrumental techniques and traditional immunoassays, particularly in terms of high sensitivity, time-saving, ease of operation, and high-throughput evaluation. Specifically, we generated an artificial antigen by chemically conjugating 3-CQA with bovine serum albumin (BSA) and produced a monoclonal antibody (mAb). Following the optimization of the reaction system, a mAb-based indirect competitive CLEIA (ic-CLEIA) was developed, with a half-maximum inhibitory concentration (IC_50_ value) of 2.97 ng/mL and a calibration range of 0.64–13.75 ng/mL, surpassing those of the conventional enzyme-linked immunosorbent assay (ELISA). Further cross-reactivity (CR) tests revealed that the established ic-CLEIA offered favorable specificity to 3-CQA. Moreover, we determined 3-CQA content in six herbs and seven patient medicine samples, with the results showing a strong correlation coefficient of 0.9667 between the ic-CLEIA and high-performance liquid chromatography (HPLC).

These findings indicate that the proposed ic-CLEIA enables rapid, simple, and environmentally friendly detection of 3-CQA. More importantly, to our knowledge, this study represents the first incorporation of ic-CLEIA into the quantification of 3-CQA, holding significant potential for ensuring the quality control of herbal products and patent medicines across various applications. Our study thus serves as an instructive example for 3-CQA determination and opens avenues for extending the application of this method to the quantification of other molecules within the CGA family.

## 2 Materials and methods

### 2.1 Preparation of artificial antigens

**Figure 2:**
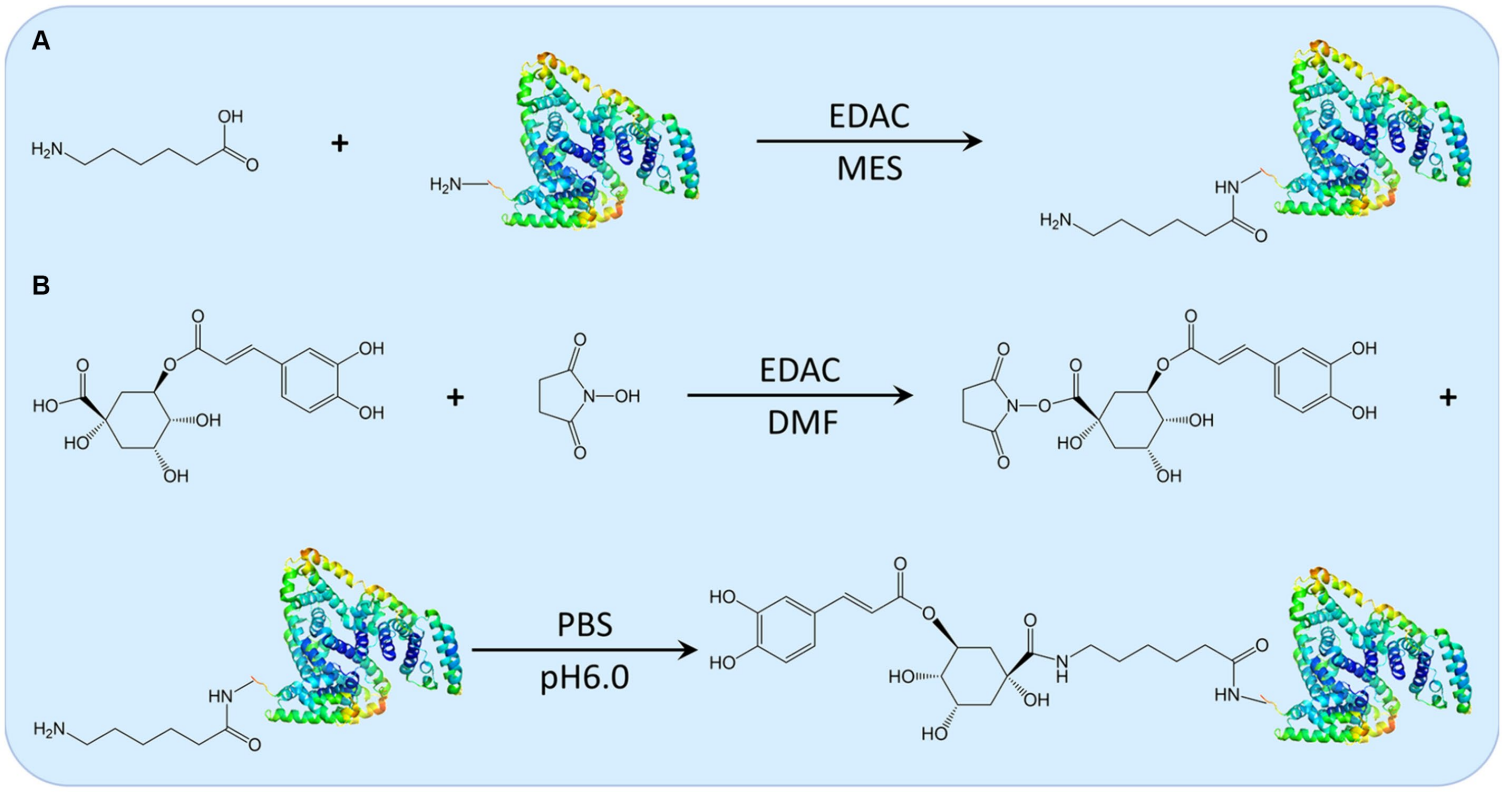
The schematic illustration of the synthesis route for 3-CQA-BSA Conjugate. Depiction of the synthesis routes for the aminated carrier protein **(A)** and the activated intermediate 3-CQA, along with the 3-CQA-BSA bio-conjugate **(B)**.

The 3-CQA artificial antigen was synthesized through the carbodiimide method (Figure 2) (18). All chemicals utilized were of analytical grade and purchased from Sigma (St Louis, MO, United States). Initially, 30 mg of either BSA or ovalbumin (OVA), 6 mg of 6-aminohexanoic acid, and 25 mg of 1-ethyl-3 (3-dimethylaminopropyl) carbodiimide (EDAC) were precisely weighted and dissolved in an 8 mL solution of 2-(N-morpholino) ethanesulfonic acid. The mixture was stirred at room temperature for 12 h. Upon reaction completion, the mixture was transferred to a dialysis bag and subjected to dialysis in phosphate-buffered saline (PBS) at 4°C for 72 h, yielding a solution of aminated carrier protein. Subsequently, 7.09 mg of 3-CQA, along with 4.38 mg of N-hydroxysuccinimide (NHS) and 3.3 mg of EDAC were weighed and dissolved in 500 μL N, N-dimethylformamide (DMF). The reaction was conducted at 17°C, 130 rpm for 16 h to generate the activated intermediate of 3-CQA. This activated intermediate was then added dropwise to the aminated carrier protein solution. Followed by stirring overnight, the mixture was dialyzed against 10 mM PBS, (pH 7.4) at 4°C for 2 days, with the dialysis solution changed four times, ensuring the removal of any unreacted 3-CQA. The resulting 3-CQA-BSA and 3-CQA-OVA bio-conjugates were finally stored at −20°C until use. Spectroscopic analysis was conducted using a Specord 50 UV–VIS spectrophotometer (Analytik AG, Jena, Germany) to confirm the successful coupling of 3-CQA to the carrier protein.

### 2.2 Generation of monoclonal antibody

The hybridoma technique was employed to produce the monoclonal antibody as follows. Six female BALB/c mice (6 to 10 weeks old) were obtained from Beijing HFK Bio-technology (Beijing, China). Following 2 weeks of adaptive feeding, the mice were immunized with equal amounts of 3-CQA-BSA (1 mg/mL, dissolved in PBS) and Freund’s complete adjuvant (Sigma, St Louis, MO, United States). Subsequently, four booster injections were administered at two-week intervals, each containing 3-CQA-BSA solution emulsified with Freund’s incomplete adjuvant (Sigma, St Louis, MO, United States). After the fifth injection, blood samples were collected from each mouse and centrifuged. The specificity of the obtained serum was determined using an indirect competitive ELISA (ic-ELISA). The positive mice were then identified, and splenocytes from these mice were fused with sp2/0 mouse myeloma cells (ATCC, Manassas, VA, United States) using PEG4000 at 37°C. Hybridomas were sequentially cultivated in HAT medium (containing hypoxanthine, aminopterin, and thymidine) and HT medium (lacking amino purine) at 37°C in a CO_2_ incubator (Thermo, Franklin, MA, United States) after fusion. After 10 days, subclone culture of surviving hybridoma cells was performed using a limited dilution method, and positive clones were detected by ic-ELISA. The identified clones were further amplified to produce ascites, which were then purified using the ammonium sulfate precipitation method (19). Following dialysis against H_2_O for 48 h, the mAb was lyophilized. The purity and concentration of purified mAb were determined by sodium dodecyl sulfate polyacrylamide gel electrophoresis (SDS-PAGE) and a BCA Protein Assay Kit (Sangon biotech, Shanghai, China), respectively. The isotype of mAb was identified using a mouse monoclonal antibody isotyping kit (Sino Biological, Beijing, China).

All the procedures described above complied with the Chinese Regulations of Laboratory Animals and were approved by the institutional experimental animal ethics committee (Chinese-German Joint Institute for Natural Product Research, Shaanxi University of Technology, and Approval Number 2021–01).

### 2.3 Optimization of working buffer and establishment of the standard curve for ic-CLEIA

To establish the optimal conditions for the ic-CLEIA, a checkerboard titration was employed. The protocols of the ic-CLEIA were performed as previously (20). A gradient concentration of 3-CQA-OVA (0.125, 0.5, 2.0, and 8.0 μg/mL; 100 μL/well) was diluted in PBS and added to a 96-well white microliter plate (Costar Inc., Cambridge, MA, United States), followed by incubation at 4°C overnight. After washing each well three times with PBST (PBS solution containing 0.05% Tween-20, pH 7.4), the plates were blocked with 5% skimmed milk powder (SMP) (Sigma, St Louis, MO, United States) in PBS (200 μL/well) at 37°C for 2 h. Subsequently, different concentrations of mAb (1:2000, 1:4000, 1:8000, 1:16000, 1:32000, 1:64000, and 1:128000; 100 μL/well) were added to different wells, followed by incubation at 4°C for 50 min. For ic-CLEIA standard curve, 3-CQA standard solutions in PBS (0.01, 0.025, 0.05, 0.1, 0.25, 0.5, 1, 2.5, 5, 10, 25, 50, and 100 ng/mL; 50 μL) and the diluted antibody in PBS (50 μL) were added to each well and incubated at 4°C for 50 min. After a washing step, goat anti-mouse IgG-horse radish peroxidase (HRP) (SinoBiological, Beijing, China) dissolved in PBS supplemented with 2% SMP was added at a 1:5000 dilution (100 μL/well), and the plates were incubated at 37°C for 50 min. Afterward, 100 μL of chemiluminescent substrate solution (Heliosense Biotechnology, Xiamen, China) was pipetted into each well, and the emitted photons were immediately read at 595 nm using a Tecan Infinite F200 reader (Mannedorf, Switzerland). To investigate the effects of different factors on the overall reaction system, different pH (6.5, 7.0, 7.4, and 8.0), methanol aqueous solution (5, 10, 30, and 50%; v/v), Tween-20 (0.01, 0.02, 0.04, 0.06, and 0.08%, v/v), sodium chloride solution (0.1 M, 0.2 M, and 0.4 M) were prepared and tested.

All incubation steps were performed in the dark throughout the procedure. The competitive inhibition curves for 3-CQA were plotted using GraphPad Prism 8.0 (GraphPad Inc., San Diego, CA).

### 2.4 Establishment of the standard curve for ic-ELISA

The protocols of ic-ELISA are similar to the ic-CLEIA, 3-CQA-OVA antigen, anti-3-CQA mAb, and goat anti-mouse IgG-HRP were successively incubated under optimized conditions. 3-CQA standard solutions in PBS (1, 2, 4, 8, 16, 32, 64, 128, 256, and 512 ng/mL; 50 μL) and the diluted antibody in PBS (50 μL) were added to each well and incubated at 4°C for 50 min. Subsequent to four washes, 3, 3′, 5, 5′-tetramethylbenzidine (TMB, 100 μL/well) solution was added and incubated at 37°C for 10 min. The reaction was then terminated by adding 2 M H_2_SO_4_ (50 μL/well), and the absorbance was measured at the wavelength of 450 nm (OD_450_). The competitive inhibition curve was plotted using Lg[10*C_3-CQA_ (ng/mL)] as the abscissa and B/B_0_ (B_0_ is the OD_450_ value without standard, and B is the OD_450_ value with standard present) as the ordinate.

### 2.5 Cross-reactivity assay

To evaluate CR, the structural analogs of 3-CQA including 4-CQA, 5-CQA, 3,5-dicaffeoylquinic acid, 4,5-dicaffeoylquinic acid, 3,4-dicaffeoylquinic acid, and tea polyphenols (DESITE Biotech, Chengdu, China) were used in place of 3-CQA within the ic-CLEIA assay. The respective CR values of these analogs were determined using the following equation:


$$CR\;\left(\%\right)=IC50\;\left(3-CQA\right)/IC50\;\left(3-CQA\;\mathrm{analogues}\right)\times 100\%\text{.}$$

### 2.6 HPLC analysis

HPLC determination of 3-CQA was performed using a Dionex Ultimate 3,000 UHPLC system (Thermo Fisher Scientific, Waltham, MA, United States). The method was established with reference to the Chinese Pharmacopeia (21). Briefly, 3-CQA solutions were prepared by dissolving in 50% methanol at different concentrations (25, 50, 100, 200, 400, and 800 μg/mL). Subsequently, the 3-CQA solutions were separated using a Shimadzu Intersil ODS3-C18 column (reversed-phase, 150 × 4.6 mm, 4.0 μm) with a mobile phase system consisting of acetonitrile and 0.4% (v/v) phosphoric acid aqueous. The remaining parameters were set as follows: column temperature = 30°C, injection volume = 10 μL, and detection wavelength = 327 nm (Supplementary Table S1), and the retention time of 3-CQA was 11.2 min. The standard curve of 3-CQA-HPLC was plotted based on the retention time of the 3-CQA reference. It was then used to extrapolate the amount of 3-CQA in the herbs and patent medicines.

### 2.7 Sample preparation and extraction of 3-CQA

Samples of six herbs (*Honeysuckle*, *Eucommia*, *Dandelion*, *Houttuynia*, *Virgate wormwood*, and *Lonicera* stem), seven Chinese patent medicines (*Shuang-Huang-Lian* capsule, Vitamin C *Yin-Qiao* tablet, *Yin-Zhi-Huang* granule, *Yin-Huang* granule, *Kou-Yan-Qing* granule, *compound Jin-Yin-Hua* granule, and *Jin-Sang-Zi* lozenge) were purchased from local pharmacies and supermarkets in Hanzhong, Shaanxi, China. The extraction of 3-CQA was conducted following the procedure prescribed in the Chinese Pharmacopeia (21). Each herb (0.1 g) and patent medicine (0.5 g) were ground into powder and dissolved in 50% methanol (5 mL). The resulting solutions were then subjected to sonication for 30 min at 250 W. After sonication, the extracts were weighed, and the lost weight was replenished with 50% methanol, followed by centrifugation at 4000 rpm for 5 min. The whole process was carried out under protected conditions to avoid exposure to light. The obtained supernatant was directly employed for immunoassay analysis after dilution. For HPLC analysis, the supernatant was further filtered through a 0.22 μm cellulose membrane prior to analysis.

### 2.8 Recovery analysis

A total of six samples, comprising three herbs and three patent medicines, were prepared in triplicate. For each sample, 0.5 mg and 1.0 mg of 3-CQA were added, leaving one sample remaining without the addition of 3-CQA as a control. The 3-CQA in the resulting mixture was extracted, followed by appropriate dilution for subsequent HPLC and ic-CLEIA analyses. Each spiked sample was measured four times in parallel. The spiked recoveries were calculated using the following formula:


$$\begin{array}{l} \mathrm{Recovery}\;\left(\%\right)=\left(\mathrm{CMeasured}\;\left(\mathrm{mg}/g\right)-C3-CAQ\;\mathrm{in}\;\mathrm{unspiked}\;\mathrm{samples}\;\left(\mathrm{mg}/g\right)\right)\\ /\mathrm{CSpike}\;\left(\mathrm{mg}/g\right)\times 100\%\text{.} \end{array}$$

## 3 Results

### 3.1 Characterization of the artificial antigen and antibody

**Figure 3:**
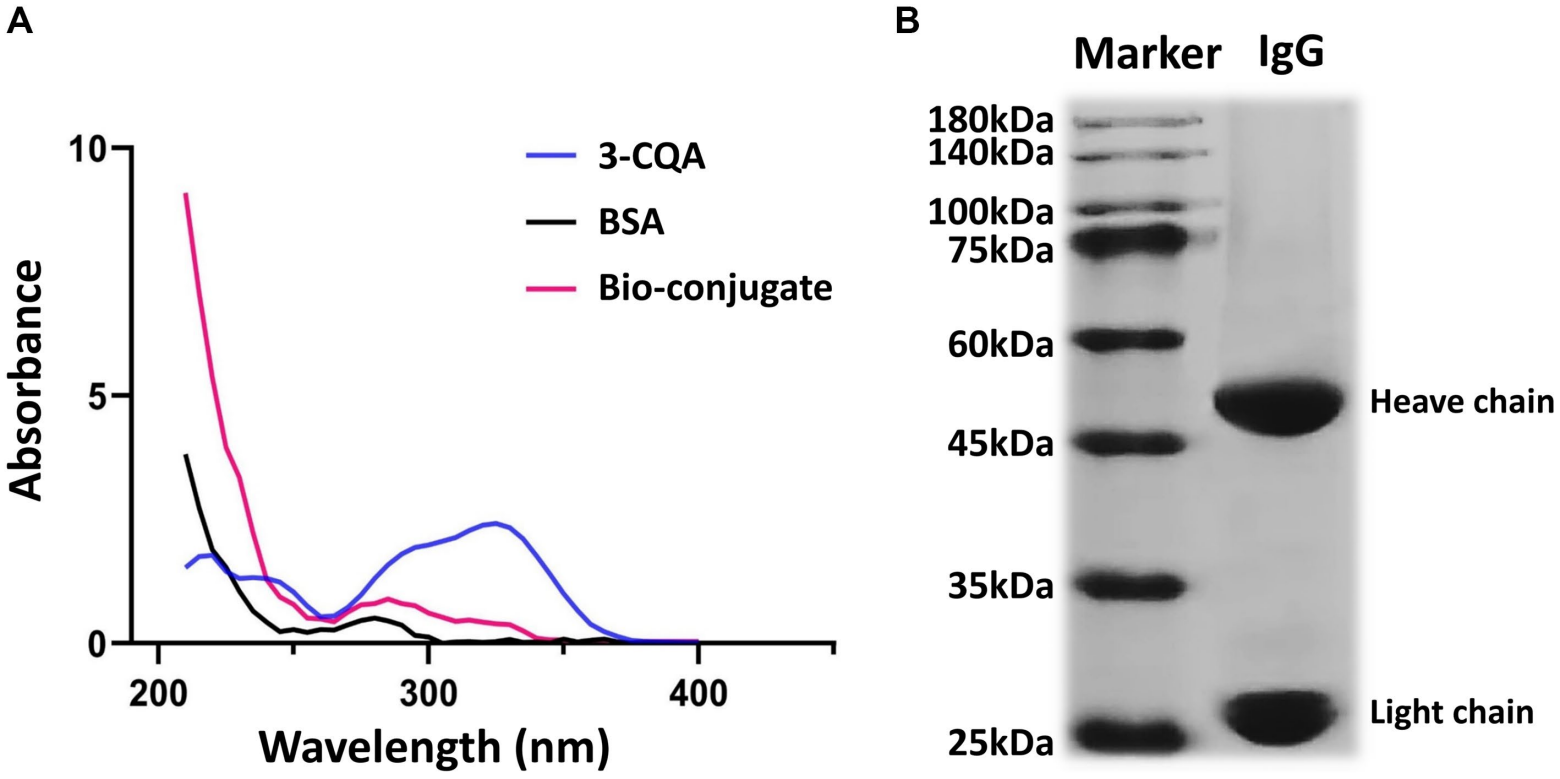
Characterization of the hapten and anti-3-CQA mAb. **(A)** Ultraviolet spectrometry analysis of 3-CQA, BSA, and 3-CQA-BSA bio-conjugate. **(B)** SDS-PAGE analysis of the mAb.

The characteristic peaks of BSA and 3-CQA were observed at 280 nm and 326 nm, respectively. In contrast, the characteristic absorption peaks of the 3-CQA-BSA bio-conjugate were identified between 280 and 326 nm (Figure 3A), demonstrating the successful synthesis of the complete artificial antigen. Likewise, the absorption peak of 3-CQA-OVA was shifted compared to that of 3-CQA and OVA (data not shown), indicating that the conjugated antigen 3-CQA-OVA was also successfully synthesized.

Regarding the antibody against the 3-CQA-BSA, the antiserum titer of the immunized mice was determined to be 1: 160000 using the ic-ELISA (Supplementary Figure S1). Subsequent purification of mouse ascites yielded two distinct bands corresponding to the heavy (50 kDa) and light chains (25 kDa) on SDS-PAGE (Figure 3B), affirming the high purity of the obtained antibodies. The purified mAb was then concentrated to 3.12 mg/mL and was classified as an IgG1 isotype with a kappa light chain.

### 3.2 Optimization of ic-CLEIA condition

**Figure 4:**
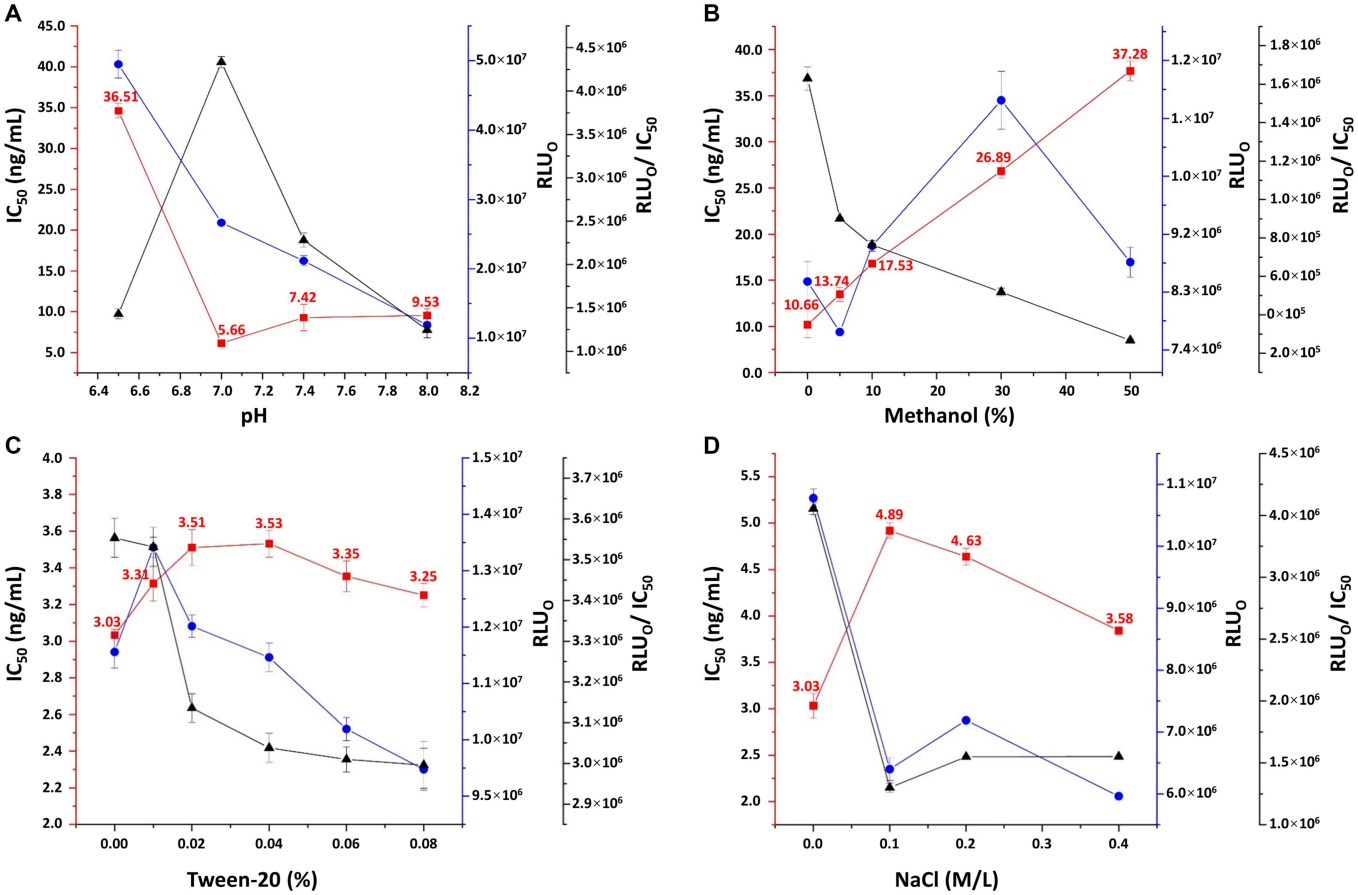
Optimization of pH **(A)**, methanol concentration **(B)**, Tween-20 concentration **(C)**, and sodium chloride concentration **(D)** in working buffer. RLU_0_: relative light unit without competing reagents. Each value represents the average of four replicates.

The relative light unit (RLU) value decreased sharply with the antibody titer from 1:2000 to 1:8000, and the trend gradually flattened as the titer reached 1:16000 (Supplementary Figure S2). Additionally, when the coating concentration of 3-CQA-OVA was below 2 μg/mL, the RLU exhibited a sharp decline (Supplementary Figure S2). Consequently, an antibody dilution of 1:8000 (0.39 μg/mL) and a coating concentration of 2 μg/mL were deemed appropriate conditions for ic-CLEIA. Regarding the pH conditions, both slightly acidic and slightly alkaline environments resulted in an increase in the IC_50_. Conversely, a neutral pH led to minimum IC_50_ while the RLU_0_/IC_50_ ratio reached the maximum (Figure 4A). Therefore, pH = 7.0 was selected as the optimal pH value. The impact of surfactants (Tween-20) and organic solvents (methanol) on the reaction system was also investigated. The IC_50_ values displayed a continuous increasing trend with higher concentration of methanol and Tween-20 added (Figures 4B,C). As such, deionized water in the absence of surfactants and organic reagents was chosen as the optimal reaction condition. Moreover, the effect of salt concentration on ic-CLEIA was explored. As shown in Figure 4D, the IC_50_ value progressively decreases with the increase of salt ion concentration in the range of 0.1–0.4 M, and reached the minimum value at 0.4 M. In case of the absence of salt ions, the IC_50_ value was distinctly lower than the case of the added salt ions, with a maximum value of RLU_0_/IC_50_ value. With comprehensive consideration, deionized water without Na^+^, surfactant, and organic reagent was selected as the working buffer, and the pH value was adjusted to 7.0.

### 3.3 Establishment of enzyme-linked immunoassay method

**Figure 5:**
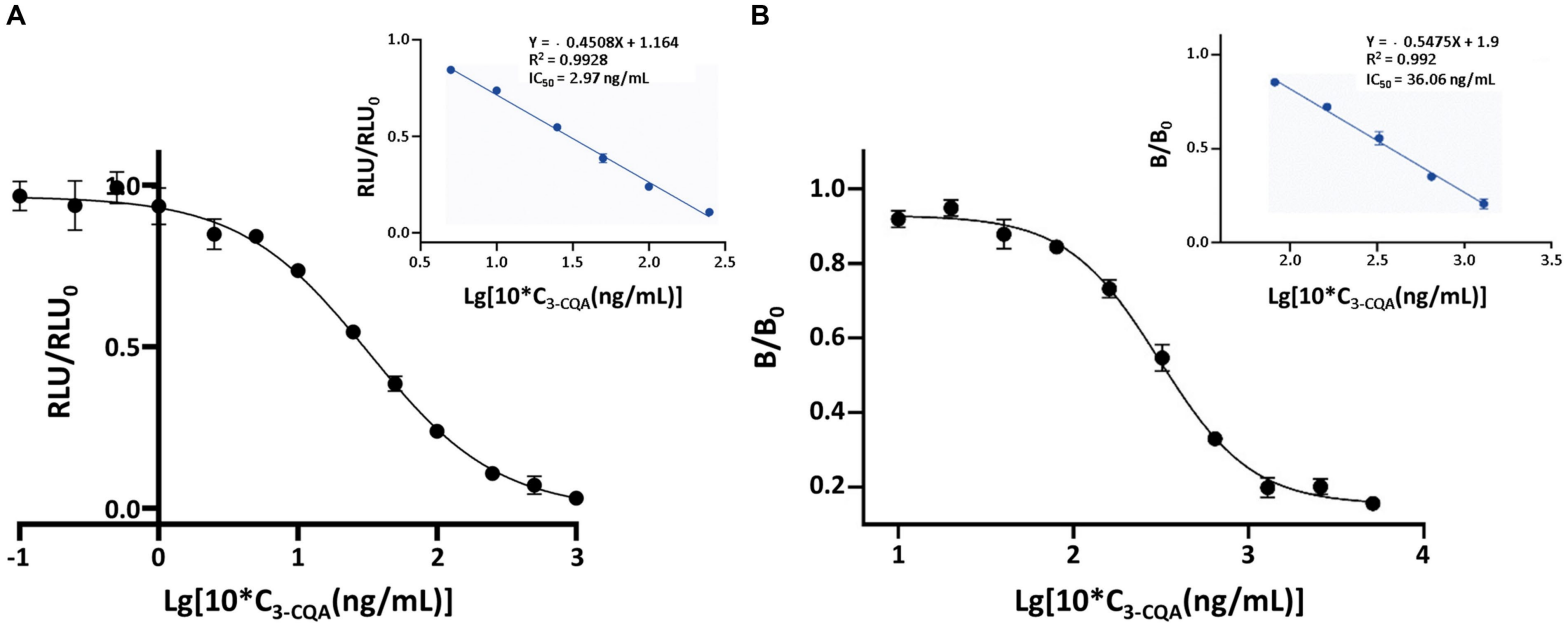
Standard curves of the ic-CLEIA **(A)** and ic-ELISA **(B)** for 3-CQA. RLU and RLU_0_ mean the relative light unit with and without competing reagents, respectively. B and B_0_ mean the absorbance value with and without competing reagents, respectively. Each value represents the average of four replicates.

Under the optimized conditions, representative competitive inhibition curve revealed an IC_50_ value of 2.97 ng/mL and the limit of detection (LOD, IC_10_) value of 0.39 ng/mL (Figure 5A). The linear range was from 0.64 ng/mL (IC_20_) to 13.75 ng/mL (IC_80_). An ic-ELISA method for detecting 3-CQA was established under the same conditions as ic-CLEIA, with a linear range of 10.21–127.36 ng/mL, an IC_50_ value of 36.06 ng/mL, and a LOD value of 6.71 ng/mL (Figure 5B).

### 3.4 Cross-reactivity

4-CQA, 5-CQA, 3,5-dicaffeoylquinic acid, 4,5-dicaffeoylquinic acid, and 3,4-dicaffeoylquinic acid were subjected to ic-CLEIA and their IC_50_ values were determined sequentially. The CR was 0.62, 1.81, 10.71, 0.31, and 2.10%, respectively (Table 1).

**Table 1:** Cross-reactivity for 3-CQA and its structural analogs.

Compound	Structure	IC_50_ (ng/mL)	CR (%)
3-CQA	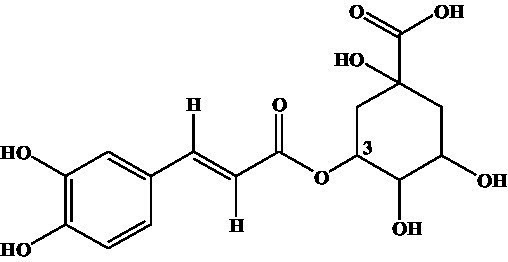	2.97	100.00
4-CQA	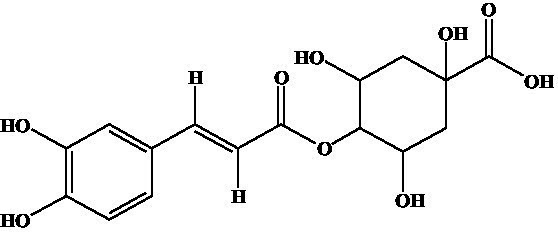	480.62	0.62
5-CQA	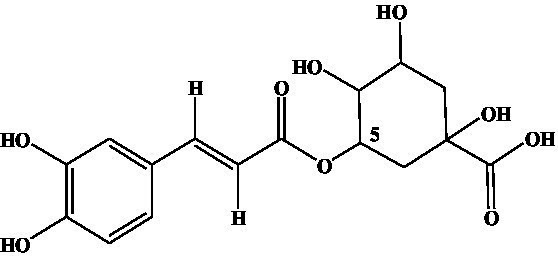	164.44	1.81
3,5-dicaffeoylquinic acid	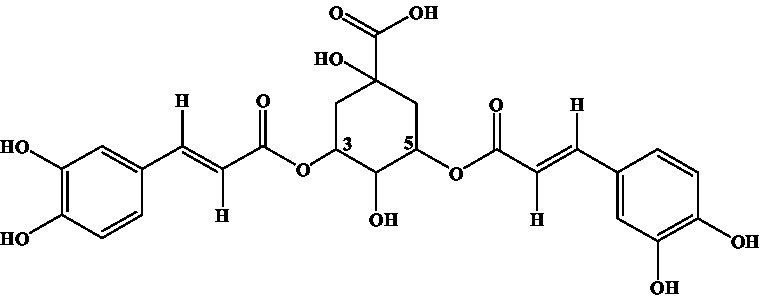	27.74	10.71
4,5-dicaffeoylquinic acid	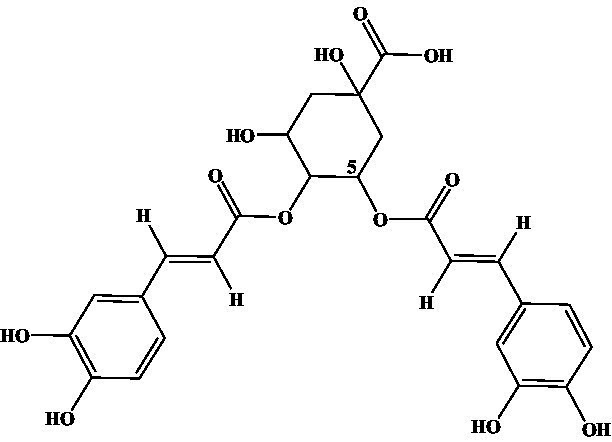	971.56	0.31
3,4-dicaffeoylquinic acid	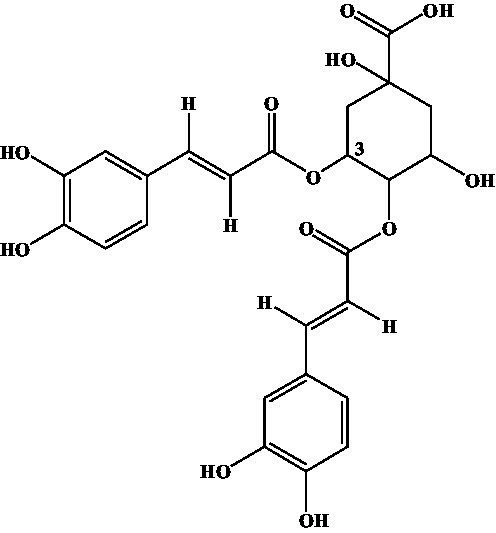	141.73	2.10

### 3.5 High-performance liquid chromatography analysis of 3-CQA

A reversed-phase HPLC was developed for 3-CQA determination, and the retention time was 11.2 min (Supplementary Figure S3). The regression curve equation was Y = 0.5494X + 4.744 (*R*^2^ = 0.9999, *n* = 4; Supplementary Figure S4).

### 3.6 Real and spiked samples detection

3-CQA was extracted from herbs, and patent medicines samples, and the 3-CQA content was determined using the established ic-CLEIA method. The 3-CQA contents of *Honeysuckle* and *Eucommia* were significantly higher than that of other herbs. *Shuang-huang-lian* capsules contain about 1.5% (w/w) 3-CQA, the highest content among the patent medicines (Table 2).

**Table 2:** Content of 3-CQA in real and spiked samples (*n* = 4).

Samples	Spiked (mg/g)	ic-CLEIA			HPLC		
		Measured (mg/g)	Recovery (%)	RSD^a^ (%)	Measured (mg/g)	Recovery (%)	RSD^a^ (%)
*Lonicera* stem	/	2.28	/	0.21	2.78	/	0.06
*Virgate wormwood*	/	0.74	/	0.05	1.05	/	<0.01
Houttuynia	/	0.62	/	0.02	0.89	/	0.03
*Kou-Yan-Qing* granule	/	5.20	/	0.32	3.68	/	0.03
Compound *Jin-Yin-Hua* granule	/	1.52	/	0.13	1.64	/	<0.01
*Yin-Zhi-Huang* granule	/	1.80	/	0.03	1.06	/	0.01
*Jin-Sang-Zi* Lozenge	/	0.27	/	0.03	0.29	/	0.01
*Dandelion*	0	1.33	/	/	1.04	/	/
	5	7.17	115.57	0.33	5.77	94.56	0.76
	10	9.64	83.06	1.90	9.64	85.99	0.87
*Honeysuckle*	0	18.37	/	/	21.31	/	/
	5	23.60	104.60	12.39	26.43	102.35	0.24
	10	29.76	113.90	0.09	30.61	92.97	0.37
*Eucommia*	0	11.91	/	/	12.44	/	/
	5	17.36	105.77	3.10	16.91	89.34	0.59
	10	22.36	112.53	6.60	21.09	86.49	0.08
*Shuang-Huang-Lian* capsule	0	12.80	/	/	12.74	/	/
	5	18.59	115.85	5.29	18.09	106.99	0.47
	10	20.39	75.87	0.41	21.75	90.10	2.47
Vitamin C *Yin-Qiao* tablet	0	8.50	/	/	4.44	/	/
	5	14.80	121.70	0.93	9.58	102.83	0.38
	10	19.64	111.44	3.64	13.99	95.45	0.06
*Yin-Huang* granule	0	1.96	/	/	2.45	/	/
	5	5.76	75.99	10.90	7.06	92.06	0.43
	10	10.50	92.45	2.97	12.60	101.43	0.21

^a^Relative standard deviation.

3-CQA standard was added to herb and patent medicines to obtain specific concentration (5 or 10 mg/g). The recovery of 3-CQA after extraction was determined by the ic-CLEIA or HPLC. The ic-CLEIA showed recoveries ranging from 75.87 to 121.70%, and the relative standard deviation was 0.09 to 12.39% (Table 2).

### 3.7 Comparison of ic-CLEIA and HPLC for detection of 3-CQA

The results of the ic-CLEIA method showed good correlation with those of HPLC (R^2^ = 0.9667), indicating that the method developed could achieve reliable and accurate determination of 3-CQA in samples (Supplementary Figure S5).

## 4 Discussion

3-CQA, a prominent member of the CGA family, exhibits a broad spectrum of bioactive and therapeutic functions (8, 22, 23). Its widespread distribution in edible foods and herbs (24, 25), coupled with the prevalence of non-standard practices in its determination, underscores the pressing need for the development of a robust quantification method. In this pursuit, we sought to establish a highly sensitive and specific ic-CLEIA method for 3-CQA analysis, as reports have yet to explore the application of ic-CLEIA for determining 3-CQA levels prior to this study.

The inherent small molecule nature of 3-CQA renders it devoid of immunogenicity. We devised a method wherein 3-CQA was chemically conjugated with BSA or OVA to generate an artificial antigen capable of eliciting an immune response in mice. Leveraging the mAb specifically targeting the conjugate, we developed an ic-CLEIA with remarkable efficacy. It demonstrated an IC_50_ of 2.97 ng/mL, displaying linear absorbance values across a concentration range of 0.64–13.75 ng/mL. Impressively, the LOD of the ic-CLEIA was 0.39 ng/mL, comparable to the reported LOD (0.1 ng/mL) using ELISA (26), while significantly outperforming the ic-ELISA (6.71 ng/mL) in our parallel investigations. In addition, in comparison to previously reported instrumental methods for 3-CQA analysis (Table 3), including HPLC, reverse-phase rapid resolution liquid chromatography, capillary zone electrophoresis technique, and near-infrared spectroscopy, our ic-CLEIA emerged as a superior alternative reflected in the aspects such as high sensitivity and avoidance of expensive equipment and specialized operations. While Zhang’s ic-ELISA demonstrated a detection range of 0.10–1.51 ng/mL, which is narrower than our ic-CLEIA range of 0.64–13.75 ng/mL, our ic-CLEIA method provides a broader detection range to accommodate a wider variety of sample concentrations.

**Table 3:** Methods developed in chlorogenic acid analysis.

Methods	Characteristics	Samples	Detection range	Reference
High-performance liquid chromatography (HPLC)	Precise; sample needs to be derivatized	Brazilian green propolis	3.75–22.5 μg/mL	(27)
Reverse-phase rapid resolution liquid chromatography	High sensitivity, precise	Green coffee samples	12.33–143.50 μg/mL	(28)
Gas chromatography–mass spectrometry	Wide range of applications, efficient	Coffee samples	Not mentioned	(29)
Spectrophotometric assays	Simple equipment, relatively large error	*Chamerionangustifolium L.*	Not mentioned	(30)
Capillary zone electrophoresis technique	Convenient, fast, economical and reliable	Tobacco residues	3–500 μg/mL	(31)
Near-infrared spectroscopy	Fast, easy operation, efficient	*Flos Mume*	20.51–1312.94 μg/mL	(32)
Flow injection chemiluminescent	High sensitivity, fast	Fruits (apple, grape, hawthorn)	50 ng/mL–50 μg/mL	(33)
Colloidal gold-based immunochromatographic assay	Fast, easy operation, visual	*Flos Lonicerae Japonicae*	>100 ng/mL	(34)
ic-ELISA	Fast, feasible, high sensitivity and specific	*Flos Lonicerae Japonicae*	0.10–1.51 ng/mL	(26)
ic-CLEIA	Fast, feasible, high sensitivity and specific	Herbs and patent medicines	0.64–13.75 ng/mL	Current study

Moreover, in terms of specificity, the established ic-CLEIA revealed relatively low cross-reactivity (<5%) with most 3-CQA structural analogs, with only 3, 5-dicaffeoylquinic acid exhibiting slightly higher cross-reactivity (10.71%) (Table 1). This occurrence implies the active role of the substitution of hydroxyl moiety in position 3 of the quinic acid in influencing cross-reactivity patterns. These results suggest the relatively high specificity of the ic-CLEIA method in determining 3-CQA. Moreover, the spiked recoveries, a critical index for evaluating the reliability of immunoassays (35), ranged from 75.87 to 121.70%, with relative standard deviations varying from 0.09 to 12.39%, thereby affirming the capability of the ic-CLEIA for detecting 3-CQA in herbs and patent medicines.

One thing worth highlighting is the eco-friendliness and user-friendly attributes of our detection process. Through optimization, we uncovered that the deionized water in the absence of ions, surfactants, and organic reagents serves as an optimal buffer for ic-CLEIA. The exclusion of organic solvents and the elimination of complex sample pre-treatment present clear advantages over HPLC and other instrumental techniques. Meanwhile, in this study, we set the interaction time at 50 min; considering the vulnerability of 3-CQA and other CGA family compounds, further optimization of reaction time may enable even greater efficiency in simultaneous detection of 3-CQA, realizing the rapid high-throughput quantification.

Furthermore, the established ic-CLEIA was applied for the analysis of 3-CQA contents in 13 herb or patent medicine samples, revealing varying levels of 3-CQA across different samples (Supplementary Table S2), with higher contents observed in *Honeysuckle* and *Eucommia*. According to the Chinese Pharmacopeia, the minimum effective contents of 3-CQA in *Honeysuckle*, *Eucommia*, and *Lonicera* stem are 1.5% (15 mg/g), 0.08% (0.8 mg/g), and 0.1% (1 mg/g), respectively (21). Consequently, the herbs in this study met the criteria for qualified 3-CQA content. In addition, these herbs with therapeutic effects as individual components are essential raw materials in patient medicines, such as *Lianhua Qingwen* capsule, renowned for their antipyretic, antibacterial, and anti-inflammatory effects (36). We tested a total of 7 patent medicines, five of which adhere to clear 3-CQA content regulations in pharmacopeia: *Shuang-huang-lian* capsules (≥ 3 mg/capsule), *Yin-huang* granules (≥ 5 mg/bag), *Yin-Zhi-huang* granules (≥ 1.8 mg/bag), *Kou-yan-qing* granules (≥ 4 mg/bag), and Vitamin C *Yin-qiao* tablets (≥ 1.5 mg/tablet) (21). Detailed information on these medicines (Supplementary Table S2) confirms their compliance with the minimum 3-CQA content requirements. Additionally, good linearity was observed between the ic-CLEIA and HPLC analysis in detecting 3-CQA (R^2^ = 0.9667, Supplementary Figure S5), further attesting to the reliability and efficiency of our developed ic-CLEIA for 3-CQA analysis.

Of course, although herbal medicine quality control mandates active ingredient detection, it is equally essential to achieve real-time and rapid detection of toxic and harmful substances generated or retained during herbal growth and processing. Our study serves as a compelling paradigm for 3-CQA determination, opening avenues for extending the application of this method to quantify other members within the CGA family, like 4-CQA, contributing to advancements in herbal medicine analysis and quality assurance.

## 5 Conclusion

Collectively, we established an ic-CLEIA with a lower IC_50_ value (2.97 ng/mL) and a wider detection range (0.64–13.75 ng/mL) compared to the ic-ELISA (IC_50_: 36.06 ng/mL; detection range: 10.21–127.36 ng/mL). Without complex sample pre-treatment steps, this novel approach was validated under different sample conditions, including herbs and patent medicines. The recoveries were satisfactory (75.87–121.70%) with RSD of 0.09 to 12.39%. The results were strongly correlated with HPLC analysis (*R*^2^ = 0.9667). In conclusion, the established method was simple, time-saving, sensitive and efficient for rapid detection of 3-CQA.

## Data Availability

The original contributions presented in the study are included in the article/Supplementary material, further inquiries can be directed to the corresponding author/s.
