# Supplementary material for: Development of an ic-CLEIA for precise detection of 3-CQA in herbs and patent medicines: ensuring quality control and therapeutic efficacy
Source: Front Nutr. 2024 Aug 21;11:1439287. doi: 10.3389/fnut.2024.1439287 (PMC11371738; doi:10.3389/fnut.2024.1439287)
Supplement: Supplementary file 1 [file Data_Sheet_1.docx]

Supplementary Material

# Supplementary Figures and Tables

## Supplementary Figures


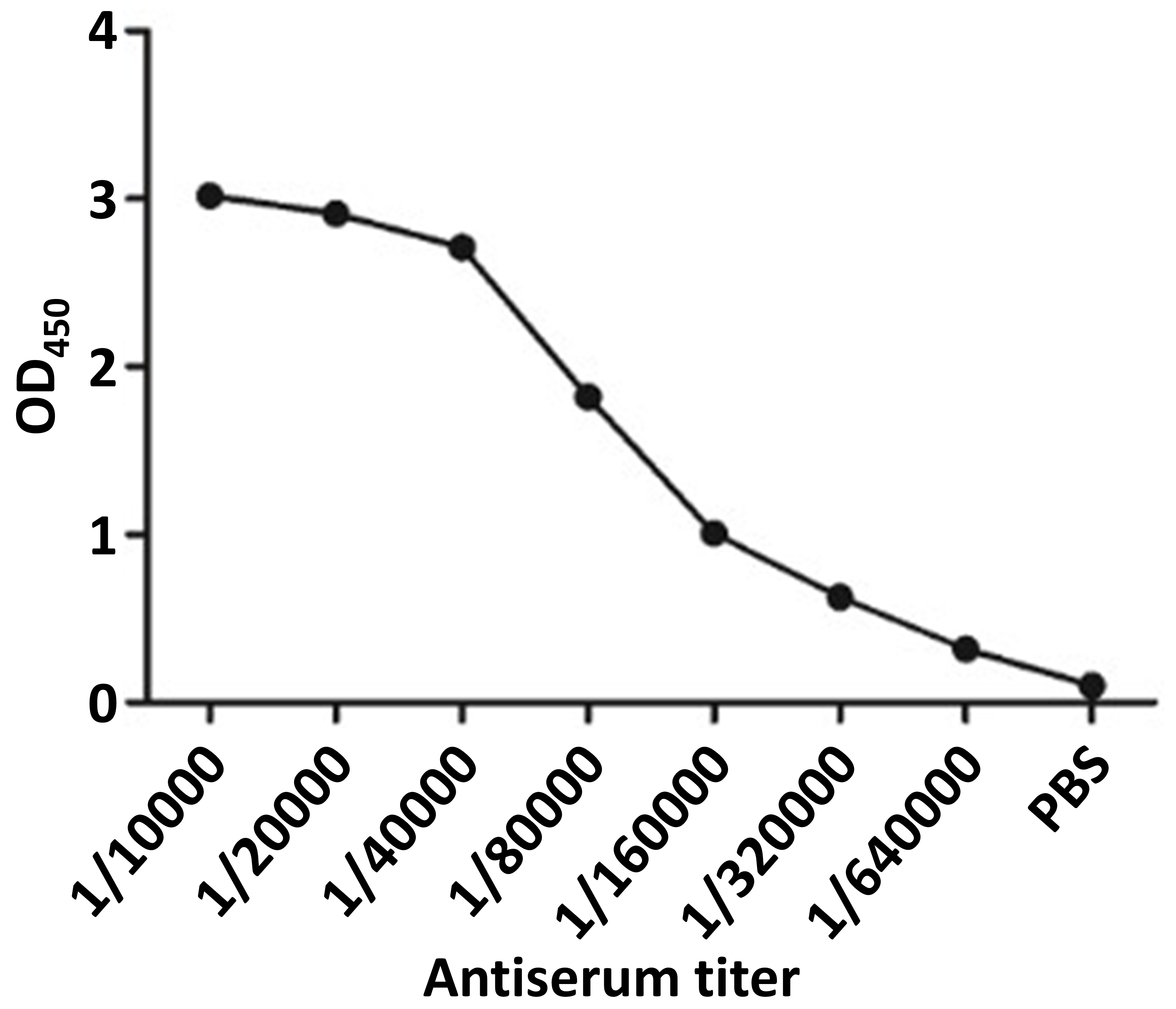


**Supplementary Figure 1.** Determination of monoclonal antibody titer in mouse serum by indirect ELISA.


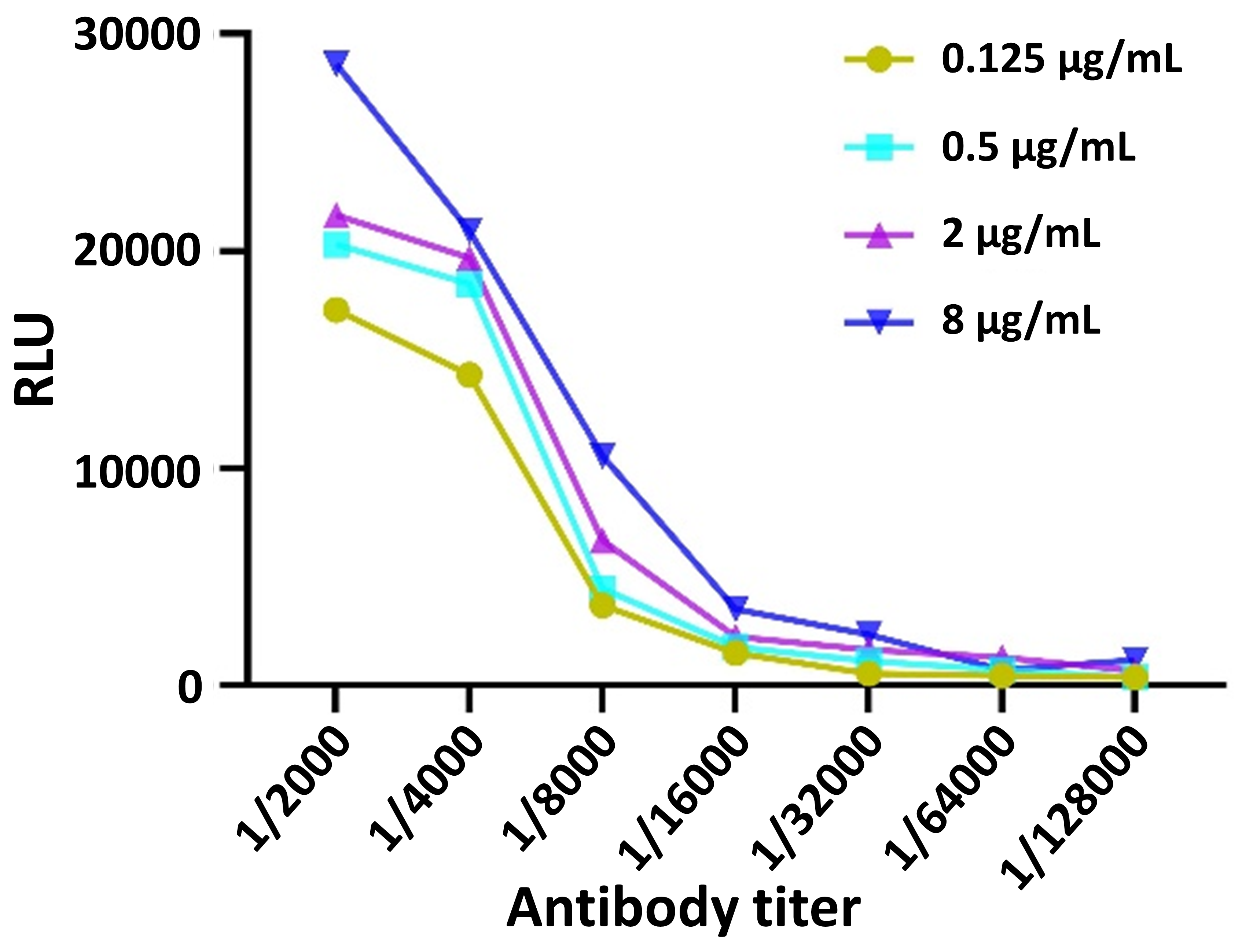


**Supplementary Figure 2.** ic-CLEIA checkerboard titration determining suitable 3-CQA-OVA. Coating concentrations ranging from 0.125 µg/mL to 8 µg/mL and mAb concentrations ranging from 0.02 µg/mL (1/128000) to 1.56 µg/mL (1/2000).


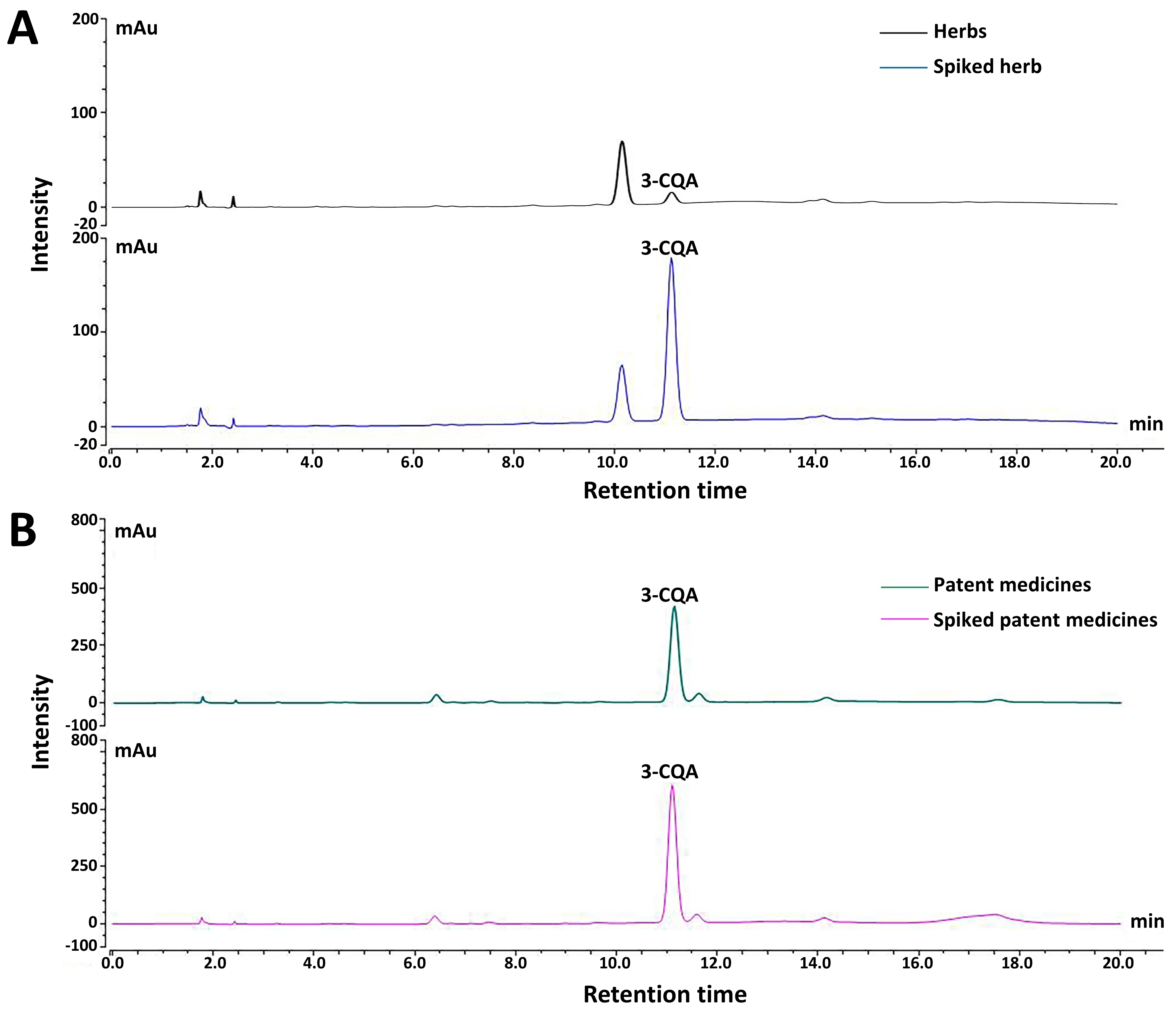


**Supplementary Figure 3.** HPLC profiles of 3-CQA spiked at 5 mg/g in herbal medicine and patent medicine. (A) Chromatographic profiles illustrating the concentrations of 3-CQA within herbal samples, with the black curve depicting the original content and the blue curve representing the herbal sample spiked with 5 mg/g 3-CQA. (B) Similarly, for patent medicines, the green curve portrays the inherent content of 3-CQA, while the red curve denotes the patent medicine sample spiked with 5 mg/g 3-CQA. The retention time of 3-CQA is 11.2 min.


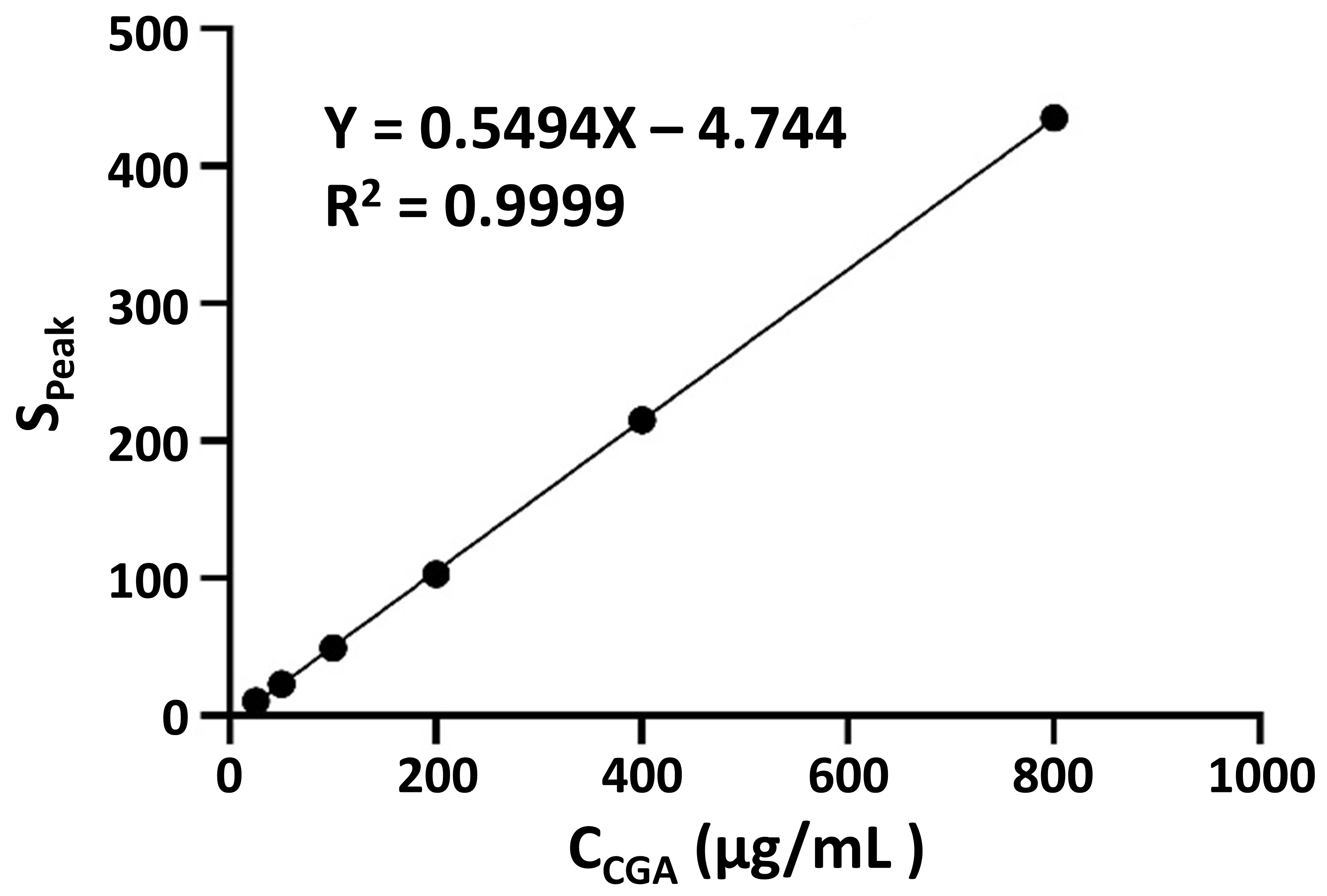


**Supplementary Figure 4.** Standard curve of the HPLC for 3-CQA.


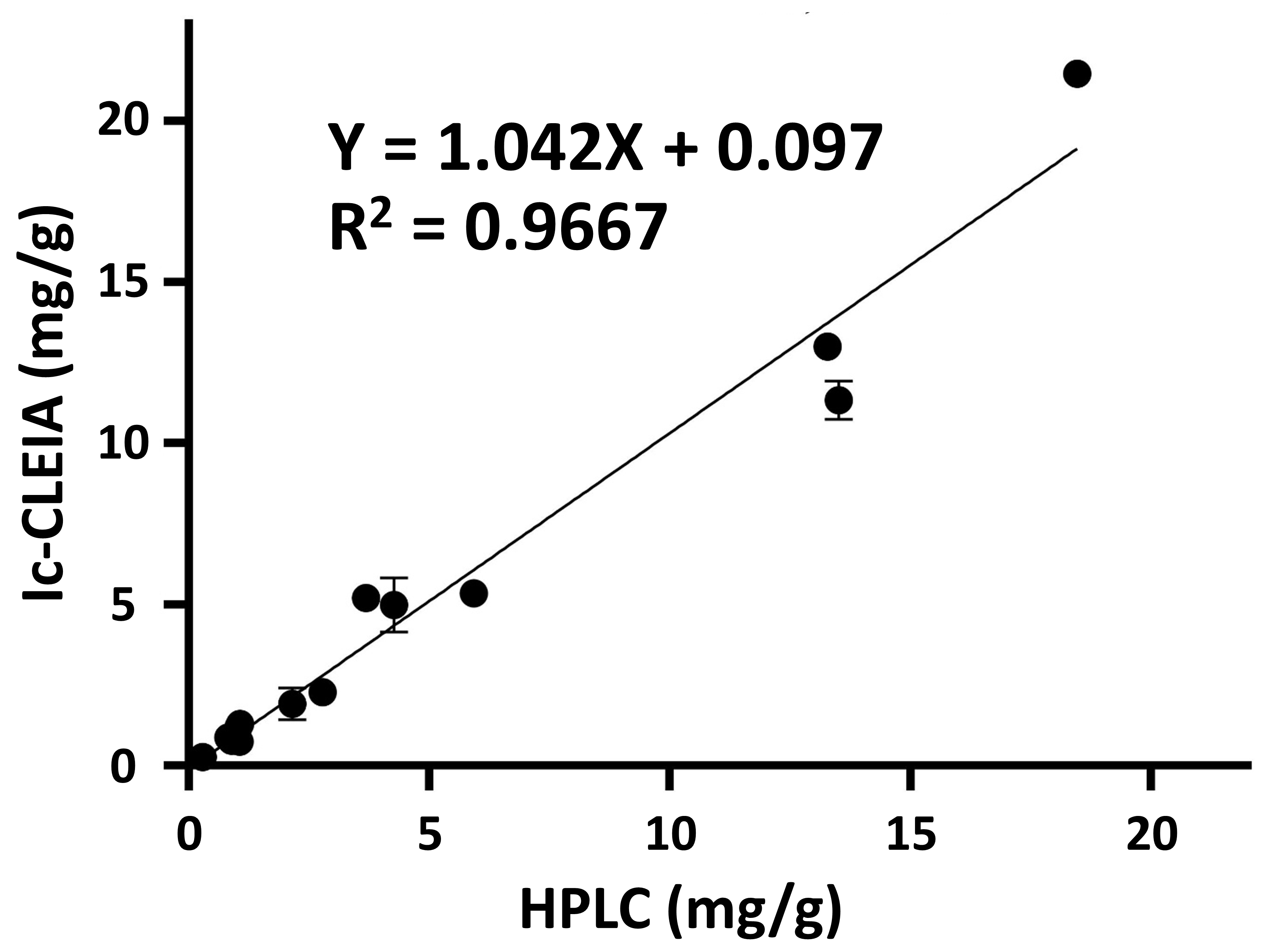


**Supplementary Figure 5.** Comparison of ic-CLEIA and HPLC for 3-CQA analysis in sample (n=4).

## Supplementary Tables

**Supplementary Table 1** Chromatographic conditions for 3-CQA

| **Time (min)** | **Mobile phase A (%)** | **Mobile phase B (%)** |
| --- | --- | --- |
| 0 | 10 | 90 |
| 2 | 10 | 90 |
| 10 | 13 | 87 |
| 15 | 13 | 87 |
| 20 | 10 | 90 |

Notes: Mobile phase A was acetonitrile absolutely and Mobile phase B was 0.4% (v/v) phosphoric acid. The flow rate was 1.0 mL/min with a running time of 20 min at 30 °C.
